# Functional Inactivation of Putative Photosynthetic Electron Acceptor Ferredoxin C2 (FdC2) Induces Delayed Heading Date and Decreased Photosynthetic Rate in Rice

**DOI:** 10.1371/journal.pone.0143361

**Published:** 2015-11-24

**Authors:** Juan Zhao, Zhennan Qiu, Banpu Ruan, Shujing Kang, Lei He, Sen Zhang, Guojun Dong, Jiang Hu, Dali Zeng, Guangheng Zhang, Zhenyu Gao, Deyong Ren, Xingming Hu, Guang Chen, Longbiao Guo, Qian Qian, Li Zhu

**Affiliations:** State Key Laboratory of Rice Biology, China National Rice Research Institute, Hangzhou 310006, China; Institute of Genetics and Developmental Biology, Chinese Academy of Sciences, CHINA

## Abstract

Ferredoxin (Fd) protein as unique electron acceptor, involved in a variety of fundamental metabolic and signaling processes, which is indispensable for plant growth. The molecular mechanisms of Fd such as regulation of electron partitioning, impact of photosynthetic rate and involvement in the carbon fixing remain elusive in rice. Here we reported a heading date delay and yellowish leaf 1 (*hdy1*) mutant derived from Japonica rice cultivar “*Nipponbare*” subjected to EMS treatment. In the paddy field, the *hdy1* mutant appeared at a significantly late heading date and had yellow-green leaves during the whole growth stage. Further investigation indicated that the abnormal phenotype of *hdy1* was connected with depressed pigment content and photosynthetic rate. Genetic analysis results showed that the *hdy1* mutant phenotype was caused by a single recessive nuclear gene mutation. Map-based cloning revealed that *OsHDY1* is located on chromosome 3 and encodes an ortholog of the At*FdC2* gene. Complementation and overexpression, transgenic plants exhibited the mutant phenotype including head date, leaf color and the transcription levels of the FdC2 were completely rescued by transformation with *OsHDY1*. Real-time PCR revealed that the expression product of *OsHDY1* was detected in almost all of the organs except root, whereas highest expression levels were observed in seeding new leaves. The lower expression levels of *HDY1* and content of iron were detected in *hdy1* than WT’s. The FdC2::GFP was detected in the chloroplasts of rice. Real-time PCR results showed that the expression of many photosynthetic electron transfer related genes in *hdy1* were higher than WT. Our results suggest that OsFdC2 plays an important role in photosynthetic rate and development of heading date by regulating electron transfer and chlorophyll content in rice.

## Introduction

Photosynthetic electron transfer chain involves excited electrons transfer via the soluble [2Fe-2S] protein ferredoxin (Fd) to NADP^+^ from photosystem I (PSI) [1]. In bacteria, algae and higher plants, Fd protein is unique electron acceptor located in the chloroplast stroma [2]. Different from cyclic electron flow, Fd, as an iron containing electron transfer factor, is able to transferring electrons to various Fd-dependent enzymes involved in photosynthesis [3,4], and also has interaction with these different enzymes in linear electron flow, either antimycin A sensitive, NADPH complex dependent, or through Fd:NADP(H) oxidoreductase (FNR) located at the cytochrome b_6_f complex [3–6]. Except the role of promoting transfer of electrons from thylakoid membrane to chloroplast stroma, Fd is supposed to be required for balancing the proportion of ATP: NADPH generated in photosynthesis [7,8]. Obviously, a variety of fundamental metabolic and signaling processes, especially photosynthesis, depend on ferredoxin proteins [2,9]. Classic ferredoxin proteins have conserved structure such as the [2Fe-2S] clusters and binding four Cys residues, and [2Fe-2S] clusters act as the redox active center making ferredoxin a powerful reductant [3]. But it is discovered that there are special ferredoxins which contain C-terminal extensions, for example FdC1 and FdC2 in *Arabidopsis* [2].

The photosynthesis taken place at the thylakoid membrane and is catalysed by embedded pigment-protein complexes, while fixation of CO_2_ occurs in the stroma. Fd as an electrons acceptor on the stromal side of the chloroplast electron transport chain, determined the efficiency of electron transfer between the thylakoid membrane and the soluble enzymes dependent on these electrons. *LOC_Os07g46460* was annotated as encoding ferredoxin-dependent glutamine: 2-oxoglutarate aminotransferase (Fd-GOGAT) and homology to *GLU1* in *Arabidopsis* whose mutant *gls1* also displayed chlorotic leaves and low chlorophyll levels [10]. Knock out *LOC_Os07g46460* and the mutant lines showed pale green leaves in rice [11]. In linear electron flow, Fds can distribute electrons from photosystem I (PSI) to Fd:NADP(H) reductase (FNR). Leaf-type FNR is a flavin adenine dinucleotide (FAD)-containing enzyme, which functions in mediating electron flow from reduced Fd to NADP^+^[3]. The yellow-green leaf mutant *502ys* showed the dropped pigment level, abnormal chloroplast development and delayed heading date. Its candidate gene *LOC_Os02g51080* encoded a FAD-binding domain containing protein and finally named *OsChlP* [12]. Double knockout of the Arabidopsis FNR genes prevents autotrophic development of the mutant plants [13], and decrease in the FNR content results in a small and pale phenotype of the plants with down-regulated photosynthetic capacity [13–17]. These data highlight the importance of Fd in chlorophyll metabolism and chloroplast development.

Furthermore, several lines of evidence suggest that Fd might not only significantly impact chlorophyll metabolism but also involved in (de)activation of a number of enzymes in carbon fixation, malate shuttling, lipid and starch metabolism, translation [18]. It is well-known that phytochromes have important funtions in mediating responses to light quality and quantity as photoperiodic photoreceptors during plant development [19, 20]. In Arabidopsis, *HY1* and *HY2* encode heme oxygenase and tetrapyrrole phytochrome chromophore phytochromobilin (PΦB) synthase, respectively, both required Fds as the electron donors to catalyze the biosynthesis of phytochromes in plastids [19, 21]. The impairment of phytochromes would impact control of floral transition, pigment synthesis and chloroplast development [20]. In rice, the *Heading date 1*-*Heading date 3a* (*Hd1*-*Hd3a*) pathway and specific *Early heading date 1*—*Heading date 3a* / *RICE FLOWERING LOCUS T1* (*Ehd1*-*Hd3α/RFT1*) pathway enable floral transition [22–24]. Moreover, photosynthetic rate can promote heading because through photosynthesis plants can accumulate enough nutrients for growth and development [25]. Decrease in the FNR content results in down-regulated photosynthetic capacity in Arabidopsis [13]. From these results, we speculate that the impairment of ferredoxins would lead to unnormal heading date for its indispensible role in the photosynthetic electron transport chain.

Nevertheless, direct evidence to relationship between ferredoxins and heading date in rice remain sparse. In this study, we isolated a **h**eading date **d**elay and **y**ellowish leaf 1(*hdy1*) mutant of rice from ethyl methane sulfonate mutagenesis. The mutant and their generations all exhibited heading date delay with yellow leaf that can be inherited stably. Map-based cloning revealed that the candidate gene *LOC_Os03g48040* encodes a 2Fe-2S iron-sulfur cluster binding domain containing protein. The protein shares 78.52% identity in acids with a ferredoxin C 2 protein in Arabidopsis. Sequencing showed a single-base mutation (G→A) at the splice site of the gene that resulted in frame shift mutation. Our results suggested Os*FdC2* plays an important role in heading date and development of rice.

## Materials and Methods

### Experimental materials

We obtain a **h**eading date **d**elay with **y**ellow leaf**1** (*hdy1*)mutant through mutagenizing the Japonica rice cultivar Nipponbare (NIP) with ethyl methane sulfonate (EMS). The F_2_ segregating population was generated by crossing the *hdy1* mutant with *indica* cv. TN1. All the plant samples were cultured under local growing conditions in experimental field of State Key Laboratory of Rice Biology, China National Rice Research Institute located in Fuyang, Zhejiang Province and Lingshui, Hainan Province.

### Investigation of important agronomic traits and Pigment measurement

Pigments were extracted from fresh leaf samples (0.15 g) at the 3-leaves stage, tillering stage and mature stage of *hdy1* and wild type plants with 10 ml of 80% acetone in the dark for 48 hours (26°C). Optical density (OD) of sample solutions was measured with an ultraviolet spectrophotometer (DU800, BECKMAN). Each sample had 3 replicates. The concentrations of chlorophyll a, chlorophyll b and carotenoid were calculated according to the methods of Lichtenthaler and Wellburn [26].

Important agronomic traits of the mutant and the wild type including plant height, effective tiller number,days to heading and thousand-grain weight were measured using 10 replicates at the mature stage. Photosynthetic rates of *hdy1* and the wild type were measured by a portable Photosynthesis system (GFS-3000, WALS) at the heading stage using 10 replicates.

### Transmission electron microscopy analysis

Leaf samples were taken from the mutant and NIP at the 3-leaves stage. The samples were placed in 2.5% glutaraldehyde (pH = 7.2) and vacuumized in a vacuum pumping machine for 30 min until the leaves sank. Samples were prepared based on the method of Kodiveri [27] and observed under a Hitachi H-7650 transmission electron microscope.

### Determination of Fe content

Leaf samples were taken from the mutant and NIP at days 55 (nine-leaf stage) after transplanting, followed by desiccation in a forced-air oven at 70°C for about 72 h to a constant weight. The desiccated samples wereground into powder. The samples were ground to pass a 0.18-mm screen mesh. About 0.5 g of dried crushed material powder was digested 2h with 8 mL of 68% HNO_3_ at 120°C, and then adds 3 mL of 30% H_2_O_2_continued digested 30mins. After cooling, the digested sample was diluted to 25 mL with deionised water. The Fe contents were determined by an ICP-emission spectrometer (Perkin Elmer Optima 2100DV). Each sample had 20 replicates.

### Marker development and map-based cloning

According to the published SSR markers and the markers designed by our laboratory (STS and INDELs), 357 pairs of markers which are uniformly distributed on 12 chromosomes were selected. Polymorphic analysis between NIP and Taizhong No.1 (TN1) was conducted, and then the identified polymorphic markers were applied in the initial location of the mutated genes. Primers were synthesized by Invitrogen (Shanghai). Each 15 μL of the reaction mixture consisted of: 20 mmol/LTris-HCl, 10 mmol/L (NH_4_)_2_SO_4_, 10 mmol/L KCl, 2 mmol/L MgCl_2_, 1% Triton X-100, pH 8.8, 0.6 U Taq enzyme, 0.17 mmol/L dNTPs, 0.33 μmol/L primers and 100 ng DNA template. Amplification was performed on an Applied Biosystems 9700 PCR system. The reaction condition was: predenaturation at 94°C for 4 min; 94°C 30s, annealing (temperatures changed with primers) for 30s, 72°C for 1 min, 30 cycles; final extension at 72°C for 10 min. The amplified products were separated by agarose gel (4%) electrophoresis, stained by ethidium bromide (EB) and observed at UV radiation.

According to the whole-genome information of rice downloaded from Gramene (http://www.gramene.org/genomebrowser/index.html) and the National Center for Biotechnology Information (NCBI) (http://www.ncbi.nlm.nih.gov/), sequence differences between Nipponbare and 9311 were analyzed by BLAST. These differences were then used to design primers. Polymorphic markers were screened and the genes related to the mutation were localized. The primers, PCR reaction system and product analysis were the same as above. The mutant (female parent) was hybridized with TN1 to construct the segregating population. The mutated individuals were selected from F_2_ generation and their DNA was extracted to identify the location of the mutated gene.

### Phylogenetic analysis

The sequences used for phylogenetic analysis were searched by NCBI BLAST. Using the OsFdC2 protein sequence as queries, TBLASTN and BLAST searches were performed on the Web pages including NCBI, the GRAMENE, and The Institute for Genomic Research (TIGR) to identify and download genomic sequences containing putative OsFdC2 orthologs. Sequences were aligned by ClustalX2 and displayed using the GeneDoc utility [28].

### Genetic complementation and over-expression test

To confirm whether the point mutation in *hdy1* is responsible for the mutant phenotype, we amplified gDNA sequence containing a 2314 bp upstream sequence, the entire coding region of *OsHDY1*, and a 1212bp downstream sequence by COM8040F: gctcggtacccggggatccATTAGTTTACGGAAAATAGATGGAC and COM8040R: aggtcgactctagaggatccAAGTCCTAAACCAAGAGAGAAGTG. This fragment was inserted into the binary vector pCAMBIA1300 for complementation test by ClonExpress® Entry One Step Cloning Kit of Vazyme Biotech and the generated transformation vector was called pHDY1. pHDY1 and control vector (pCAMBIA1300, pCK) were introduced into the *hdy1* plants via an Agrobacterium tumefaciens–mediated transformation, respectively. Hygromycin-resistant transgenic lines were selected.

The full length cDNA (552bp) of *OsHDY1* amplified by OVER8040F: gagctcggtacccggggatccATGGCTCCGTGCCCCGCCGC and OVER8040R: caggtcgactctagaggatccTCACTCATCACCCATTGCAAGCTCCAGTGC, was inserted into pCAMBIA1300S with 35S promoter to obtain the over-expression vector pOVERHDY1. Transformation was performed as described above.

### RNA Preparation and RT-PCR Analysis

Samples (200mg) were taken from different organs of NIP and *hdy1* under normal growth conditions. Total RNA were extracted using TRIzol, and corresponding cDNA were reverse transcribed using a reverse transcription kit (TOYOBO). Expressions of *HDY1* in different organs including root, stem base, 2th leaf, 3th leaf, 4th leaf, 5th leaf from seeding and leaf sheath, uppermost internode, flag leaf and panicle from heading were analyzed by real-time fluorescence quantitative PCR. The reaction system consisted of: cDNA template 1μl, SYBR Premix Ex Taq 5μl, upstream/downstream primer (10 μM) 0.3μl each, and sterile ddH_2_O 3.6μl. The reaction procedures were as follows: 95° for 5 min predenaturation; 95°10 s, 60°30 s, 72°15 s, 40 cycles. The relative expression level in each tissue was acquired by comparison with the expression of *OsACT1* gene [29]. The RT-PCR primers for the *OsHDY1* gene were re834-2F: GTGCGGTTCGGATAAAGTCA and re834-2R: AAACCAACACACAACAATGCA. The primers for *OsACT1* were CCATTGGTGCTGAGCGTTT (forward) and CGCAGCTTCCATTCCTATGAA (reverse) [29].

### Subcellular localization

To investigate the subcellular localization of HDY1 in rice, a coding region fragment lacking a stop codon was amplified from WT plants using primers GFP8040F: tatttacaattacagtcgacATGGCTCCGTGCCCCGCCGC and GFP8040R: atggatcctctagagtcgacCTCATCACCCATTGCAAGCTCCAGTGC and introduced into GFP vector pCA1301-35S-S65T-GFP by ClonExpress® Entry One Step Cloning Kitof Vazyme Biotech. Transformed the construct into rice protoplasts and detected GFP fluorescence using a confocal laser-scanning microscope (OLYMPUSIX71). The rice protoplasts prepared according to the protocols described by Yu et al [30].

### RNA-seq and Data Analysis

RNA samples were prepared from the fifth leaves of WT and *hdy1* plants, grown in 1/2 *MS* culture medium for three weeks under a 13-h light/11-h dark photoperiod at 30°C in a Versatile Environmental Test Chamber (SANYO, MLR-351) [31]. Total RNA was extracted using a Trizol kit (Takara). 50bp single end RNA-sequencing was conducted using an Illuminia Hi-seq 2500 platform from Shanghai Genergy Biotech Co. Cufflinks methods [32] were used for determination of expression values. A gene with a cut-off value of two-fold change and p-value less than 0.01 was defined as a differentially expressed gene.

## Results

### Loss of HDY1 function strongly affects heading date, photosynthetic rate and chloroplast development

**Fig 1:**
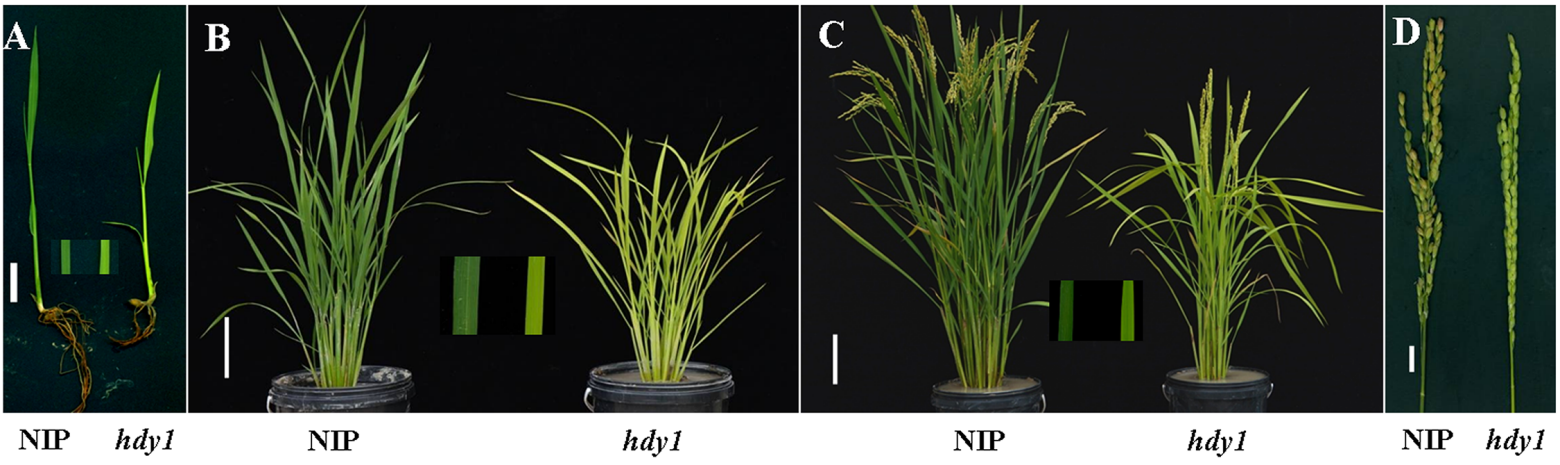
Phenotypes of NIP with *hdy1* mutant at seedling stage(A), tillering stage (B) heading stage(C), and the closeup picture of panicle (D). A and D Scale bar = 1 cm, B and C Scale bar = 10 cm.

The *hdy1* mutant was isolated from NIP subjected to EMS treatment. In the paddy field, the mutant and their generations all exhibited heading date delay with yellow leaf (*hdy1*) that can be inherited stably. Comparing to the WT, *hdy1* showed a 33-days delay in heading date and accompanied by a number of pleiotropic phenotypes, such as, yellowed leaf in the whole growth stage, reduced plant heights, the number of effective tillers, and 1000-grain weight (Fig 1; Table 1).

**Table 1:** The agronomic traits of WT and *hdy1*.

	Plant height(cm)	Days to heading (d)	No. of effective tillering	1000-grain weight(g)
**NIP**	69.89±3.29	79±3.50	15±2.00	23.28±0.08
*hdy1*	40.89±2.18**	112±3.50**	10.90±0.94**	19.87±0.71**

** Represents a significant difference between wild type and mutant at the 0.01 level.

**Fig 2:**
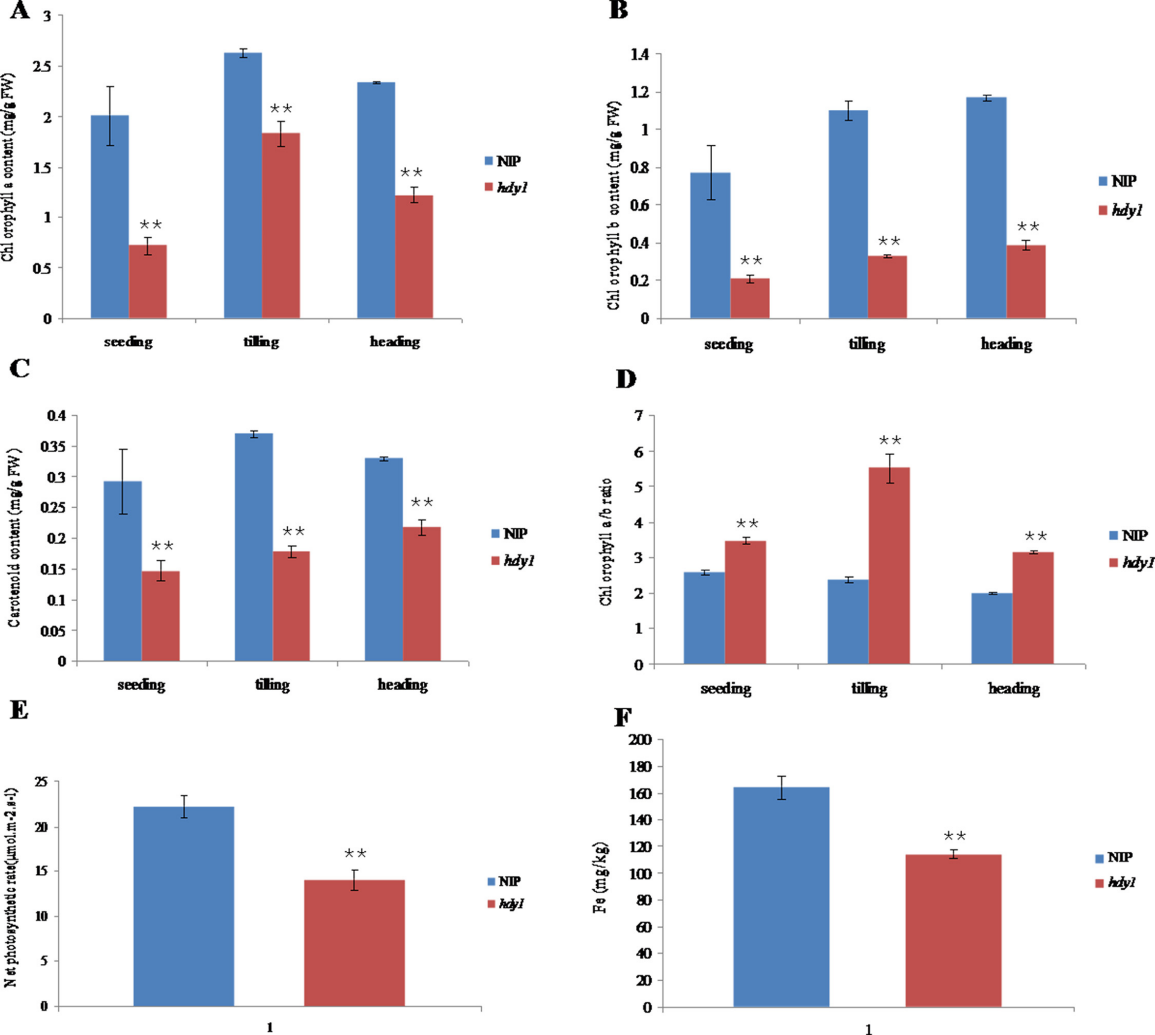
Analysis the pigment contents, net photosynthetic rate and Fe contents in the leaves of NIP and *hdy1*. (A-D) Pigment contents in the leaves of NIP and *hdy1*. Each sample had 3 replicates and 80% acetone was used as control sample. (E) Net photosynthetic rate in the flag leaves of NIP and *hdy1*. Each sample had 10 replicates. (F) Fe contents in the leaves of NIP and *hdy1* at day 55 after transplanting. Each sample had 20 replicates. **Represents a significant difference between wild type and mutant at the 0.01 level.

To characterize the mutant phenotype, we determined the chlorophyll and carotenoid contents of the leaves from the *hdy1* mutant and wild-type parent at seeding, tilling and mature stages.The results showed that contents of Chla, Chlb, and carotenoid in *hdy1* mutant plants at three stages were drastically lower than those in WT (Fig 2A–2C), whereas, the ratio of Chla/b increased in *hdy1* compared with the wild type at three stage (Fig 2D). Net photosynthetic rate was obviously decreased in flag leaf of *hdy1* compared with the wild type (Fig 2E).Our results indicate that the *hdy1* yellow leaf phenotype resulted from reduced Chla, Chlb, as well as carotenoid levels.

Based on early research findings that iron, as an essential element for Chl biosynthesis, limitation induces leaf chlorosis due to the Chl content decrease [33]. So we determine whether the reduced pigments connected with Fe content in *hdy1*, leaf samples were taken from mutant and NIP at days 55 (nine-leaf stage) after transplanting to measure the Fe content. The results showed in Fig 2F indicated that the mutant *hdy1* had the significantly dropped Fe content compared with wild type and suggested that loss of HDY1 function affects iron content in rice.

**Fig 3:**
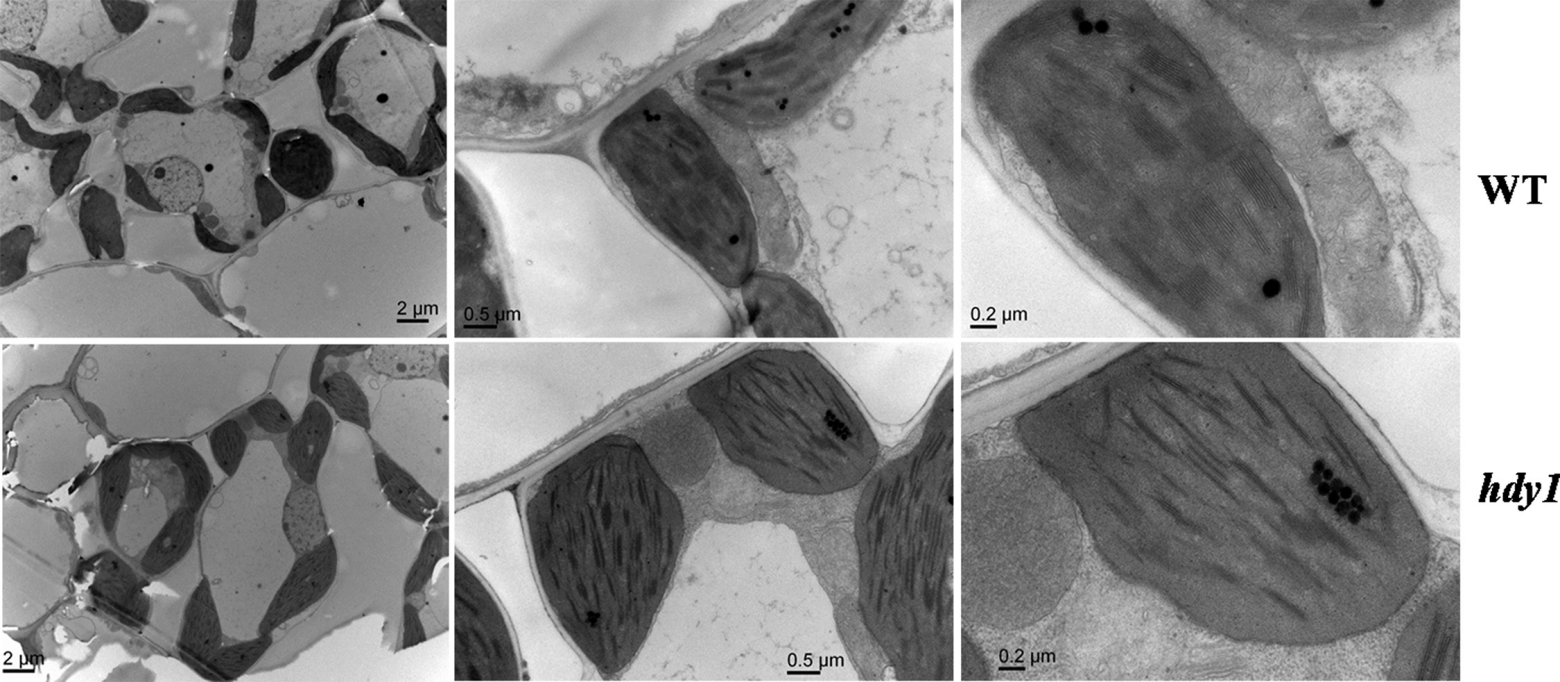
Microstructures of the NIP and *hdy1* chloroplasts under transmission electron microscope.

To confirm whether the reduced pigments accompanied by chloroplasts structure changed, we compared the ultrastructure of chloroplasts in the *hdy1* mutant and wild type at seeding stage under a transmission electron microscopy. The results showed that the number of chloroplasts in the WT and *hdy1* mesophyll cell were not obviously different, whereas the WT mesophyll cell contained densely and regularly stacked grana and the thylakoid lamella of the chloroplast in the *hdy1* mutant was abnormal and contained fewer lamella (Fig 3). Our observations revealed that *hdy1* mutation obviously affected chloroplast development.

### Map-based cloning of the *hdy1* locus and sequence analysis

To confirm the *hdy1* phenotype was decided by single-gene mutation or multiple-genes mutation, we surveyed 65 M_2_ plants of mutagenesis line *hdy1* at two weeks after germination for genetic analysis. Of these, we found that 51 plants showed the wild-type phenotype and the restshowed *hdy1* phenotype, which accorded with the 3:1 segregation ratio (÷^2^ = 0.37 < ÷^2^0.05 = 3.84). What’s more, among 350 F2 individuals generated by a cross between *hdy1* and TN1, 268 plants displayed the normal phenotype and 82 displayed the *hdy1* phenotype, which also accorded with the 3:1 segregation ratio (÷^2^ = 0.45<÷^2^0.05 = 3.84) [29]. These results above confirmed that the phenotype of *hdy1* was determined by a single recessive nuclear gene.

**Fig 4:**
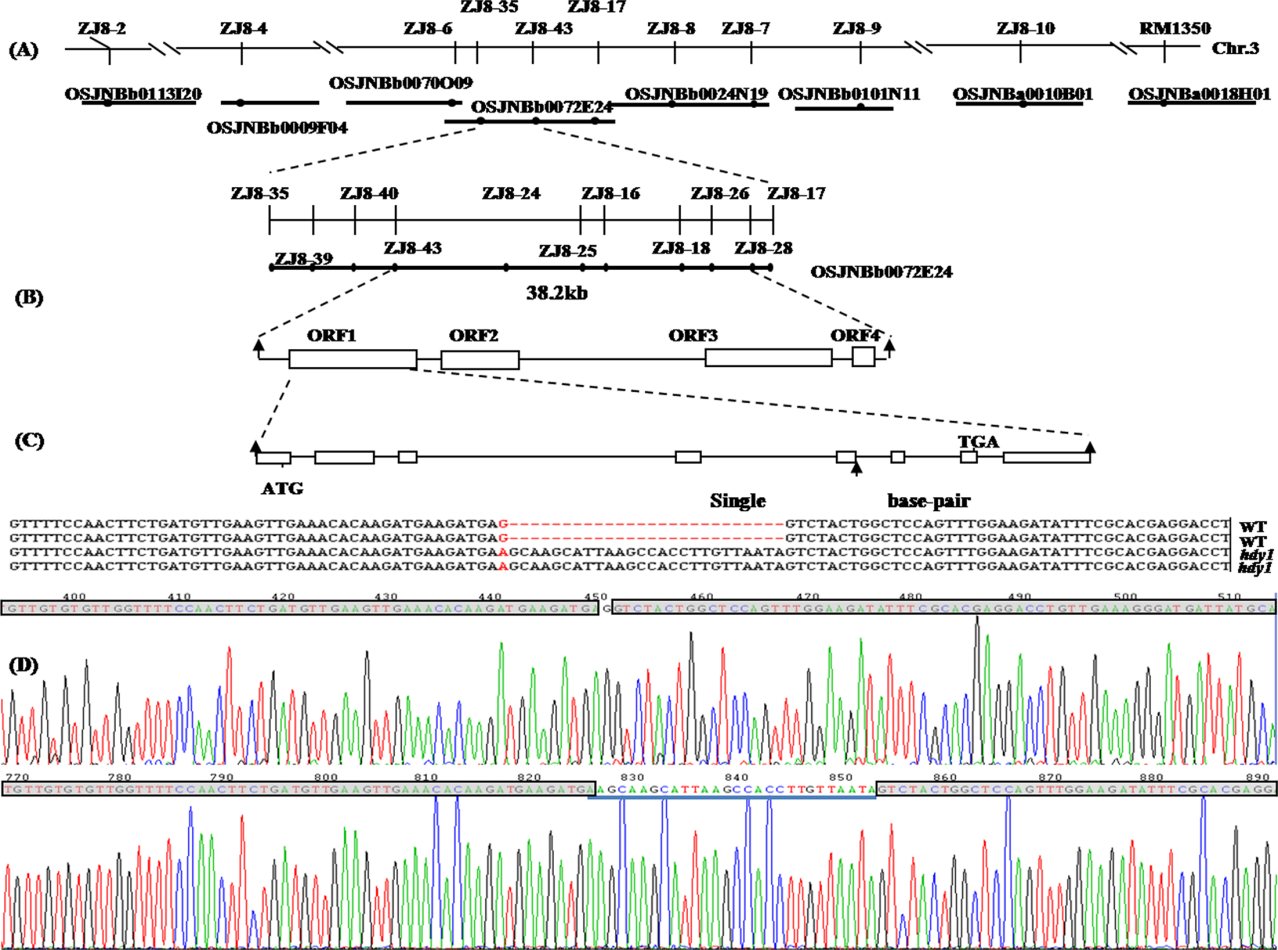
Map-based cloning and mutation site of *OsHDY1*. (A) The *OsHDY1* locus was initially mapped on chromosome 3 (Chr. 3). (B) The *OsHDY1* locus was fine mapped at a physical interval of 38.2 kb with developed markers. (C) *OsHDY1* structure and mutation site. (D) Compare sequencing diagrams of NIP and *hdy1*. The sequences of cDNA are shaded gray and the extra sequences of *hdy1* are blue underlined.

The single recessive *hdy1* locus was initially located on the long arm of chromosome 3 by using 21 *hdy1*/TN1 F2 individuals and 157 SSR and STS markers distributed in all the rice chromosomes (Fig 4A). To further improve the fine map of *hdy1*, we designed new STS markers between ZJ8-2 and RM1350 on the basis of the sequence difference between *O*. *sativa* japonica variety NIP and *O*. *sativa indica* variety 9311 (S1 Table). Eighteen pairs of primers with polymorphisms between *hdy1* and TN1 were used for the fine mapping of *HDY1*. By using more than 5400 individual genotypes, we mapped the *hdy1* locus between the two STS markers, ZJ8-43 and ZJ8-28, at a physical interval of 38.2 kb (Fig 4B). We obtained four predicted open reading frames (ORFs) in the bacterial artificial chromosome clone OSJNBb0072E24 according to the Rice Genome Annotation Project. Sequenced all ORFs and found a single-base mutation (G→A) at the splice site and 26 bps extra cDNA sequence appeared in ORF of *HDY1* that resulted in frame shift mutation and introduced a premature stop codon at 162 amino acid residue (Fig 4C and 4D).

### Complementation experiment and over-expression test

**Fig 5:**
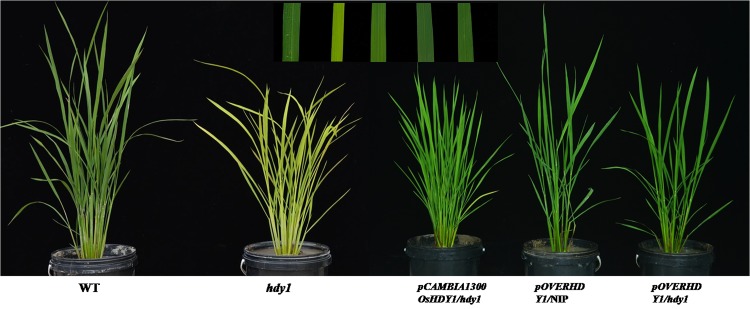
Complementation and over-expression test of *hdy1*. Transgenic lines all showed green leaf phenotypes and normal heading date.

**Fig 6:**
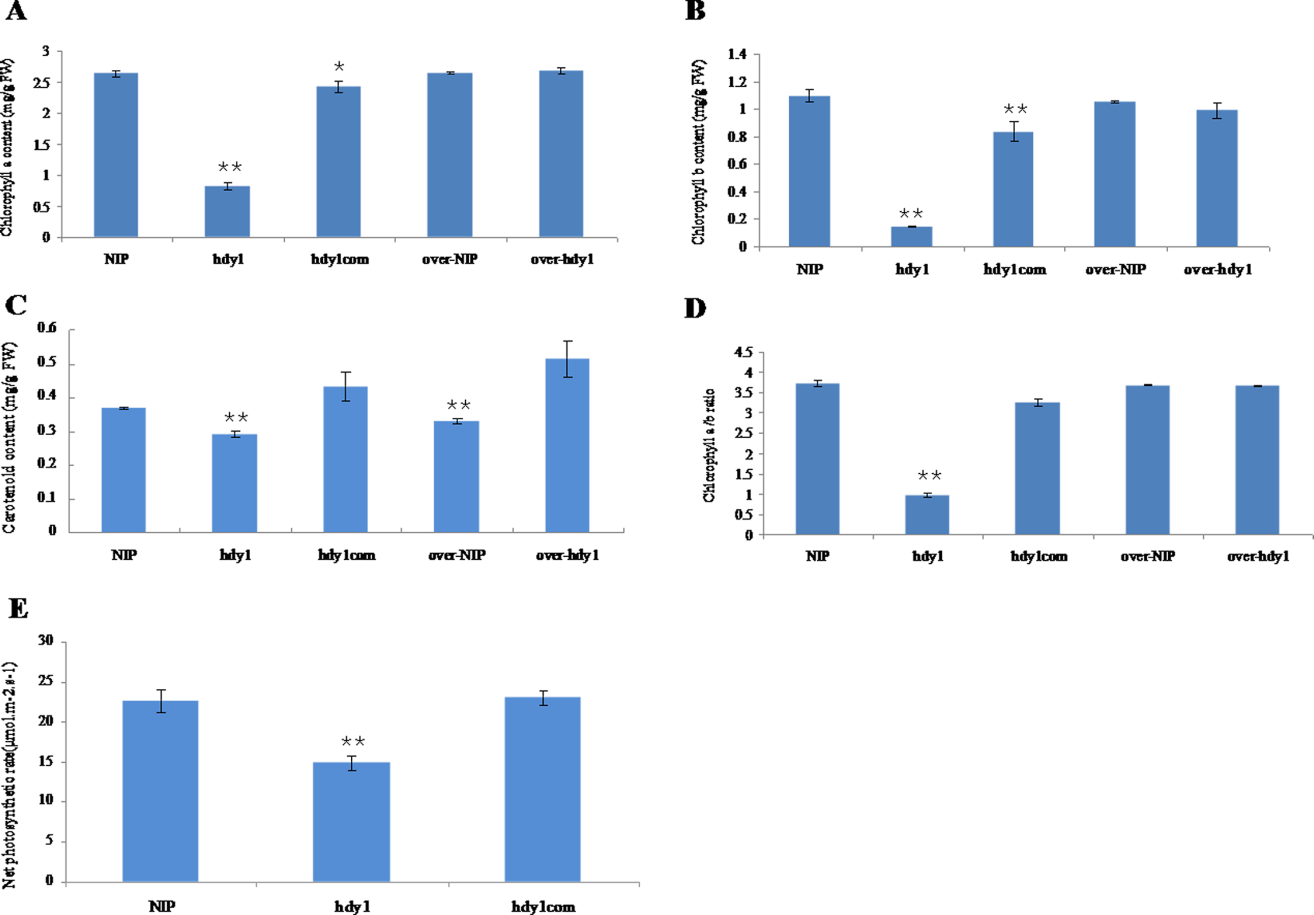
Analysis the pigment contents and net photosynthetic rate of NIP, *hdy1* and transgenic lines. (A-D) Pigment contents in the leaves of NIP, *hdy1* and transgenic lines. Each sample had 3 replicates and 80% acetone was used as control sample. (E) Net photosynthetic rate in the flag leaves of NIP, *hdy1* and transgenic lines. Each sample had 10 replicates. *and **represent a difference between wild type and mutant at the 0.05 and 0.01 level, respectively.

To confirm whether the point mutation in *hdy1* is responsible for the mutant phenotype, we constructed the complement plasmid pHDY1 containing a 2314 bp upstream sequence, the entire coding region of *OsHDY1*, and a 1212 bp downstream sequence. 45 independent transgenic lines (pHDY1/*hdy1*) completely reverted to a green leaf phenotype (Fig 5). All *hdy1* plants transformed with the empty vector (pCK) exhibited the same phenotype as that of the *hdy1* mutant (data not show). Sequenced results showed that the mutation locus of HDY1 in transgenic lines (pHDY1/*hdy1*) were heterozygous at T_0_ lines, and the wild-type phenotype and the mutant phenotype matched with the 3:1 segregation ratio at T_1_ lines. The heading date, pigment content and expression of *OsHDY1* also rescued by transformation with *OsHDY1* in *hdy1* (Figs 5 and 6). These results unambiguously indicated that the *OsHDY1* mutation is responsible for heading date delay and yellow leaf of *hdy1* (Figs 5 and 6).

**Fig 7:**
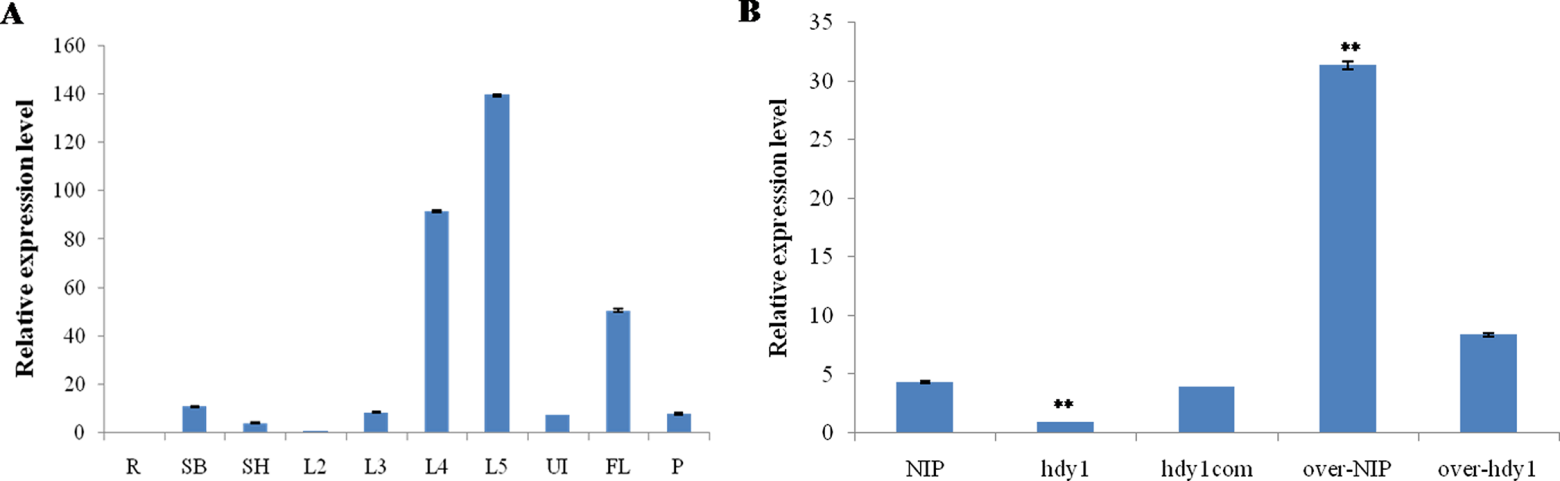
Expression levels of OsFdC2 in NIP, *hdy1* and transgenic lines. (A) Expression levels of OsFdC2 at in NIP from different organs. R: root; SB: stem base; L2-L5: 2th-5th leaf from seeding; SH: leaf sheath; UI: uppermost internode; FL: flag leaf; P: panicle from heading. (B) Expression levels of OsFdC2 in the 5th leaf from seeding of NIP, *hdy1* and transgenic lines. **represent a difference between wild type and mutant at the 0.01 level.

To further study the function of HDY1 in plant growth and development, we also constructed an over-expression plasmid (pOVERHDY1) and transformed it into the recessive mutant *hdy1* and NIP. The transgenic plants displayed normal phenotypes as well (Fig 5): the morphological characteristics intransgenic plants were rescued compared with NIP including leaf color, pigment contents and plant height (Figs 5 and 6A–6D). The transcription levels of the *HDY1* in transgenic plants were obviously higher than the mutant and WT (Fig 7B). It is suggested that the loss-of-function mutation in OsHDY1 is responsible for yellow leaf of *hdy1* and increased transcription level of *OsHDY1* does not affect visible phenotype of WT.

### FdC2 encodes a 2Fe-2S iron-sulfur cluster binding domain containing protein

**Fig 8:**
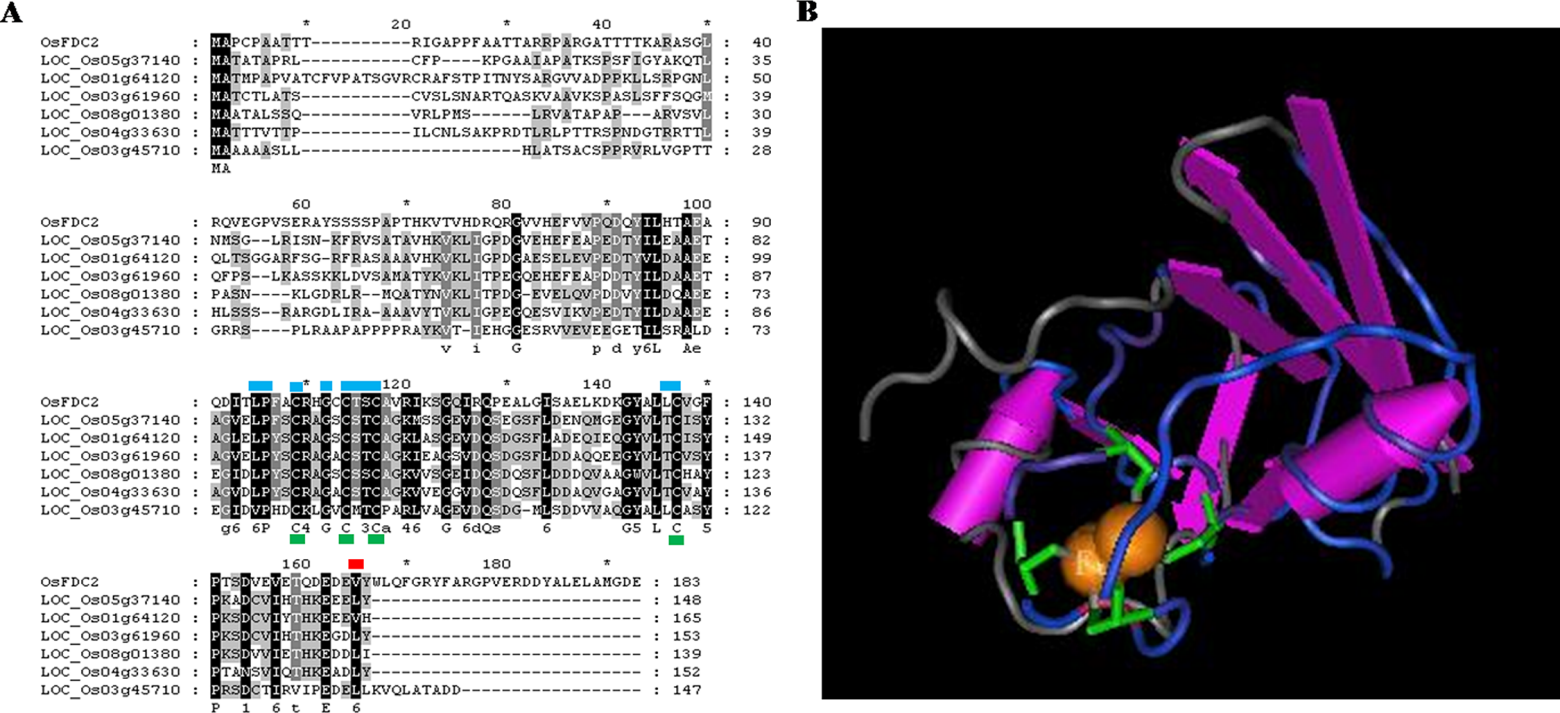
Sequence alignment and three-dimensional structure of OsFdC2. (A) Sequence analysis of OsFdC2 homologs. red flag is the mutation site of *hdy1*, blue flags are catalytic loops, green flags are four perfectly conserved iron-binding site. (B) Three-dimensional structure of FD. The green parts are Fe-binding amino acid residues. Conserved Protein Domain: cd00207, Structure:1A70.

According to the prediction of RGAP database, *LOC_Os03g48040* was annotated as encoding a [2Fe-2S] cluster binding domain containing protein, ortholog of the *FdC2* genes in Arabidopsis. Comparison of the genomic and cDNA sequences showed that *OsFdC2* consists of 7 exons and 6 introns. The *OsFdC2* ORF contains 552 bp that encodes a polypeptide of 183 amino acid residues. A 2Fe-2S iron-sulfur cluster binding protein LOC_Os03g45710 shared about 71% amino acid sequence homology with the AtFDC1. There are 12 proteins matched with 2Fe-2S iron-sulfur cluster binding protein and a ferredoxin 1-like isoform 1 protein (LOC_Os09g33950) were predicted in the rice genome (http://rice.plantbiology.msu.edu/cgi-bin/putative_function_search.pl). Sequence analysis of OsFdC2 homologs results showed that LOC_Os01g64120, LOC_Os03g61960, LOC_Os04g33630, LOC_Os08g01380,LOC_Os05g37140 and LOC_Os03g45710 shared higher amino acid sequence identity, conservative iron binding site and catalytic loops (Fig 8A). Moreover, OsFdC2 and LOC_Os03g45710 with extended C termini respectively. It is suggested that FdC1, FdC2 and at least five putative FDs are present in the rice genome. Furthermore, LOC_Os07g01930, LOC_Os09g26650, LOC_Os05g48160, LOC_Os09g33950 and LOC_Os07g30670 shared lower amino acid sequence identity and there are no conservative iron binding sites (S1 Fig). In addition, LOC_Os03g50540 was also predicted as 2Fe-2S iron-sulfur cluster binding domain containing protein, but a NADH dehydrogenase subunit G structure was found. So LOC_Os03g50540 may be a ferredoxin-NADP^+^ reductase rather than Fd homolog protein. The three dimensional structure of 2Fe-2S iron-sulfur cluster binding (cd00207 Structure: 1B9RA) was set up in Conserved Domain Database. Typically, Fds are composed of three to six β-strands and one to three α-helices, with the [2Fe-2S] cluster located at one end which contained 4 perfectly conserved iron-binding site, catalyzed electron transfer in redox reactions (Fig 8B).

### Subcellular Localization of FdC2

**Fig 9:**
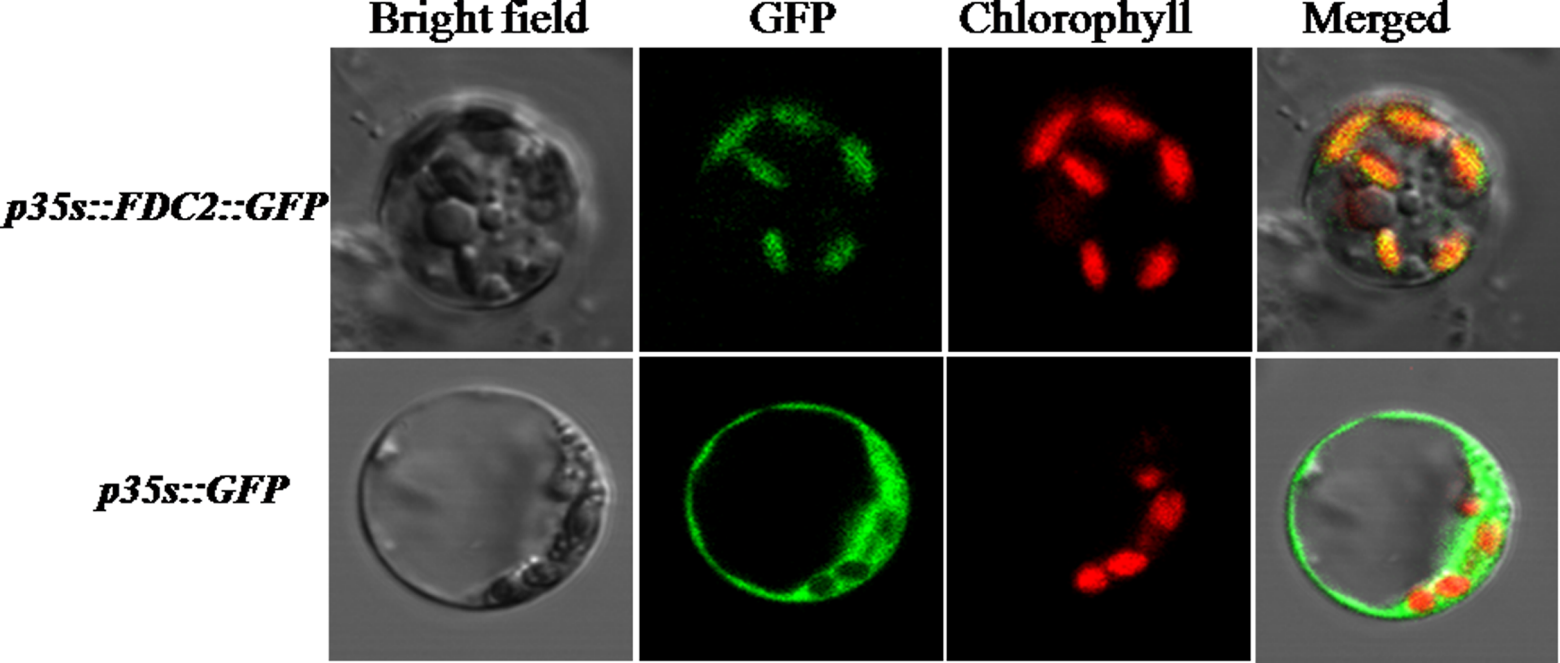
The subcellular localization of OsFdC2 protein. The subcellular localization vector contained the C-terminal amino acids of FdC2 and GFP with the 35S promoter. GFP signals of the *p35s*::*FdC2*::*GFP* fusion protein localized in the chloroplast by transformation into rice protoplasts and empty GFP vector *p35s*::*GFP* was a control. Green fluorescence showed GFP, red fluorescence showed chloroplast autofluorescence, and yellow fluorescence showed the merged fluorescence.

To determine the subcellular localization of FdC2, we constructed a pFdC2-GFP vector containing the C-terminal amino acids of FdC2 and GFP with the 35S promoter. The pFdC2-GFP plasmid and the empty GFP vector were transformed into rice protoplasts, and observed the green fluorescent signals under a confocal microscopy. The green fluorescent signals revealed that the fusion protein FdC2-GFP clearly localized to the chloroplast(Fig 9).

### Expression analyzed of the *FdC2* gene by real-time PCR

To investigate the tissue-specific expression pattern of *OsFdC2*, total RNA were extracted from different organs including root, stem base, 2th leaf, 3th leaf, 4th leaf, 5th leaf from seeding and leaf sheath, uppermost internode, flag leaf and panicle from heading. Quantitative real-time PCR analysis revealed that the expression of *FdC2* transcript can detected in all tissues and organs except root. The highest expression levels of *FdC2* were observed in the young developing leaves. Relatively lower expression levels were detected in the leaf sheath, culms and panicles but hardly detected in the root (Fig 7A). Then, we examined the transcription levels of the *FdC2* in WT, *hdy1* and three transgenic plants, hdy1com, over-WT and over-hdy1 at seedling stage by quantitative real-time PCR analysis. The results showed that expression of *FdC2* was markedly reduced compared with that of the wild-type plant in the mutant, whereas the transcription levels of the *FdC2* in hdy1com, over-WT and over-hdy1 were rescued (Fig 7B). Thus, these results indicated that the mutation of *OsFdC2*, caused to the decreased expression of *OsFdC2*, is responsible for heading date delay and abnormal leaf color in *hdy1*. The increased transcription levels of the *FdC2* rescued the phenotype of *hdy1* but do not found other visible phenotype in WT.

### FdC2 regulates expression of photosynthetic electron transport related genes

**Fig 10:**
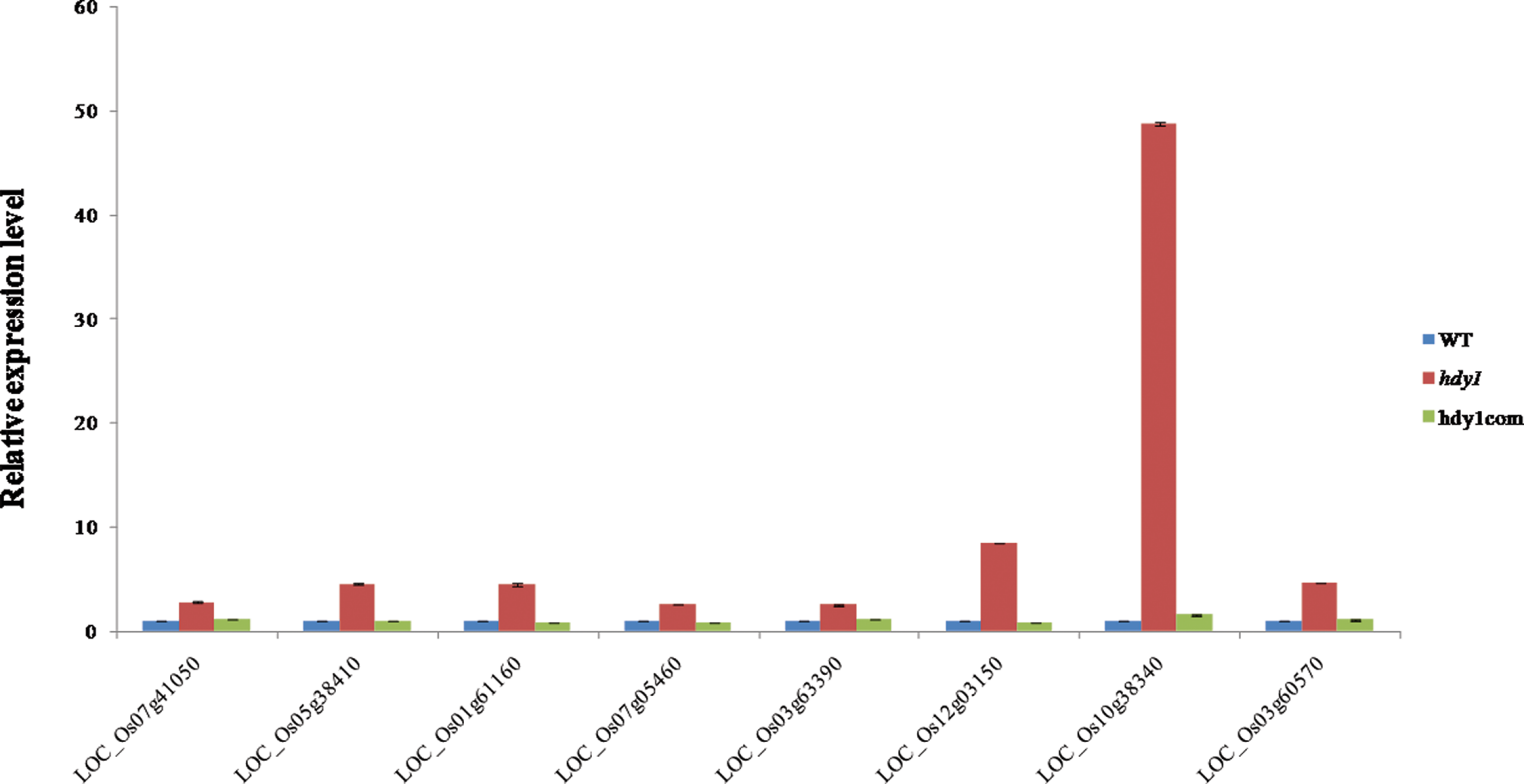
Real-time PCR to verify the expression of part of photosynthetic electron transport related genes in WT, *hdy1* and hdy1com transgenic plant. LOC_Os07g41050 (putative NAD dependent epimerase/dehydratase), LOC_Os05g38410 (putative laccase precursor protein), LOC_Os01g61160 (putative laccase precursor protein), LOC_Os07g05460 (putative flavonol sulfotransferase), LOC_Os03g63390 (putative plastocyanin-like domain containing protein), LOC_Os12g03150 (putative myb-like DNA-binding domain containing protein), LOC_Os10g38340 (putative glutathione S-transferase GSTU6) and LOC_Os03g60570 (putative ZOS3-22—C2H2 zinc finger protein) annotated by http://rice.plantbiology.msu.edu/index.shtml.

In bacteria, algae and higher plants, Fd has been shown to be involved in photosynthetic electron transfer [2]. Therefore, we examined the expression of *hdy1* in relation to electron transport related genes through RNA-seq and verified the results by real-time PCR. RNA samples were prepared from the fifth leaves of three-week-old WT and *hdy1* plants. Genes whose expressions showed at least fourfold difference between the *hdy1* and NIP were further analyzed. Besides, several genes related to electron transfer were identified. Our results indicated that three genes encoding NAD dependent epimerase/dehydratase a NADP-dependent oxidoreductase and a NADH reductase, 10 genes encoding plastocyanin-like domain containing proteins, 11 genes encoding copper metabolism related proteins and 12 genes encoding flavin/flavonol related proteins were up regulated in the *hdy1* (Fig 10). Moreover, several other metabolic route genes, such as fat and carbohydrate metabolism, rapid alkalinization factor (RALF) family protein and zinc finger protein, also were up regulated in *hdy1*. As shown in Fig 10, LOC_Os07g41050 (putative NAD dependent epimerase/dehydratase), LOC_Os05g38410 (putative laccase precursor protein), LOC_Os01g61160 (putative laccase precursor protein), LOC_Os07g05460 (putative flavonol sulfotransferase), LOC_Os03g63390 (putative plastocyanin-like domain containing protein), LOC_Os12g03150 (putative myb-like DNA-binding domain containing protein), LOC_Os10g38340 (putative glutathione S-transferase GSTU6) and LOC_Os03g60570 (putative ZOS3-22—C2H2 zinc finger protein) were all up regulated in the *hdy1*, and these genes expressions of WT and hdy1com transgenic plant were similar. It is suggest that higher expression of other electron transport related genes might to compensate less electron transfer to diverse Fd-dependent enzymes in *hdy1*. Accumulating evidence shows that reduced Fd also acts as an electron transfer in the nitrogen assimilation pathway, which is essential for plant development [34]. From our results, we suggest that FdC2 plays a pivotal role in photosynthetic rate and development of heading date might due to affected the electron transfer in rice. More biological function studies on FdC2 such as the cell signaling pathway, and transcriptional regulation in rice remains further research.

## Discussions

Photosynthesis is a top priority event for autotrophic plants, which synthesis important carbohydrates and ATP for life activities [35]. In higher plants, ferredoxin as an essential element of photosynthetic electron transport chain involved in a variety of fundamental metabolic and signaling processes [19]. In this study, we identified a heading date delay rice mutant *hdy1*. The *hdy1* mutants showed a number of abnormal phenotypes, such as heading date delay and yellow leaves, impaired development of plant height and the number of effective tillers. Map-based cloning revealed that *HDY1* encodes a [2Fe-2S] cluster binding domain containing protein, which is ortholog of the FdC2 genes in Arabidopsis and photosynthetic rate was significantly reduced in *hdy1*. From these results, we suggest that FdC2 plays a pivotal role in photosynthetic rate and chlorophyll biosynthesis in rice and closely interrelated with development of heading date, plant height and yield of rice.

Significant progress has also been made toward elucidating the linkages between photosynthetic electron transport and chlorophyll metabolism, photosynthetic capacity, such as Fd-GOGAT and OsChlP both depended on Fds for physical activities, and their mutants both displayed pale green leaves; knockout of the Arabidopsis FNR leaf senescence results in a small and pale phenotype of the plants with down-regulated photosynthetic capacity; over-expression of *OsFNR2* in Arabidopsis results in a small and pale phenotype of the plants with down-regulated photosynthetic capacity and nitrogen assimilation [11,12,34,36,37]. In rice, little is known about the molecular mechanisms of FD. As unique electron acceptor, Fd also plays a role in the nitrogen assimilation pathway via transferring electrons to Glu synthase and nitrite reductase, which is vital for plant growth and development [34]. Accumulating evidence suggests that Fd can transfer electrons to various Fd-dependent enzymes such as phytochromobilin (PΦB) synthase A, B, C and OsChlP, whose related mutants all showed abnormal heading date in rice [12,21,38,39]. What's more, Wang et al [40] demonstrated that heading date related protein Ghd7 was also a locus of natural variation in Chl content and negatively correlated the Chl content in rice. Our observations demonstrate that decreased Chl content and photosynthetic rate of *hdy1* (Figs 1–3) can lead to retarded growth and deficient nutrients for development of leaves and panicles. It is suggested that heading date delay in *hdy1* might caused by less transferred electrons during the nitrogen assimilation pathway and resulted growth slowness of plants. Over-expression of OsFdC2 results in the modification of photosynthesis and heading date (Fig 5). Interestingly, Arabidopsis plants over-expressing OsLFNR1OE and OsLFNR2OE did show growth defects and the defects phenotypes were recovered in nutrient rich culture. It may be caused that the partitions of electrons from Fd to NADP^+^ andnitrogen assimilation are disturbed by OsLFNR1OE and OsLFNR2OE. As a whole, above results indicated that the allocation proportion of Fd-transferred electrons either to photosynthesis or nitrogen assimilation is important for plants growth optimal.

The most striking function of the FdC2 is acted as an electron transfer during photosynthesis of higher plants. So it is interesting to investigate whether FdC2 mutations affected expression level of other electron transfer related gene. Real-time PCR and RNA-seq analysis results showed that the transcriptional level of several plastocyanin-like domain genes, copper metabolism and flavin/flavonol related genes were up regulated in the *hdy1*. Plastocyanin is a copper (Cu)-requiring protein and is located in the thylakoid lumen of the chloroplast where it functions as a photosynthetic electron transfer between the cytochrome b_6_f complex and PS I [41–43]. Structurally, multicopper oxidases (MCOs), at least four Cu atoms-contained enzymes, are classified into three groups according to their different spectroscopic features (T1-T3). Substrate is oxidized at T1, and then electrons are rapidly transferred to trinuclear copper cluster (TNC) through the Cys-His pathway [44–46]. Laccases belong to the MCOs family, which can oxidize a series of substrates with the four electron reduction from dioxygen to H_2_O [47–49]. Flavin-dependent monooxygenases are involved in a wide range of biological processes, and at least 130 flavin-dependent monooxygenases have been described. Group A-B flavin-dependent monooxygenases (EC 1.14.13) contain single-component enzymes that rely on NAD(P)H as external electron donor [50]. From these results, we suggest that FdC2 plays a pivotal role in electron transfer during photosynthesis of higher plants and closely interrelated with heading date regulation, net photosynthetic rate, Fe metabolism and chlorophyll biosynthesis regulation of rice.

In conclusion, we have conducted a study on a late heading date and yellow leaf mutant *hdy1* of rice. The mutation was caused by a basesubstitution in gene *OsFdC2*, which encodes a ferredoxin protein possessing a [2Fe-2S] cluster. Although the molecular mechanisms that underlie between mutant phenotypes and FdC2 linkages remain elusive, these initial findings have motivated us to re-examine the physiological implications of FD function in rice.

## Supporting Information

S1 Fig Sequence alignment OsFdC2 and other putative 2Fe-2S iron-sulfur cluster binding domain containing proteins. (TIF)

S1 Table Makers for map-based cloning of *OsFdC2*. (DOCX)
