# Supplementary figures and images for: Functional Inactivation of Putative Photosynthetic Electron Acceptor Ferredoxin C2 (FdC2) Induces Delayed Heading Date and Decreased Photosynthetic Rate in Rice
Source: PLoS One. 2015 Nov 24;10(11):e0143361. doi: 10.1371/journal.pone.0143361 (PMC4657970; doi:10.1371/journal.pone.0143361)

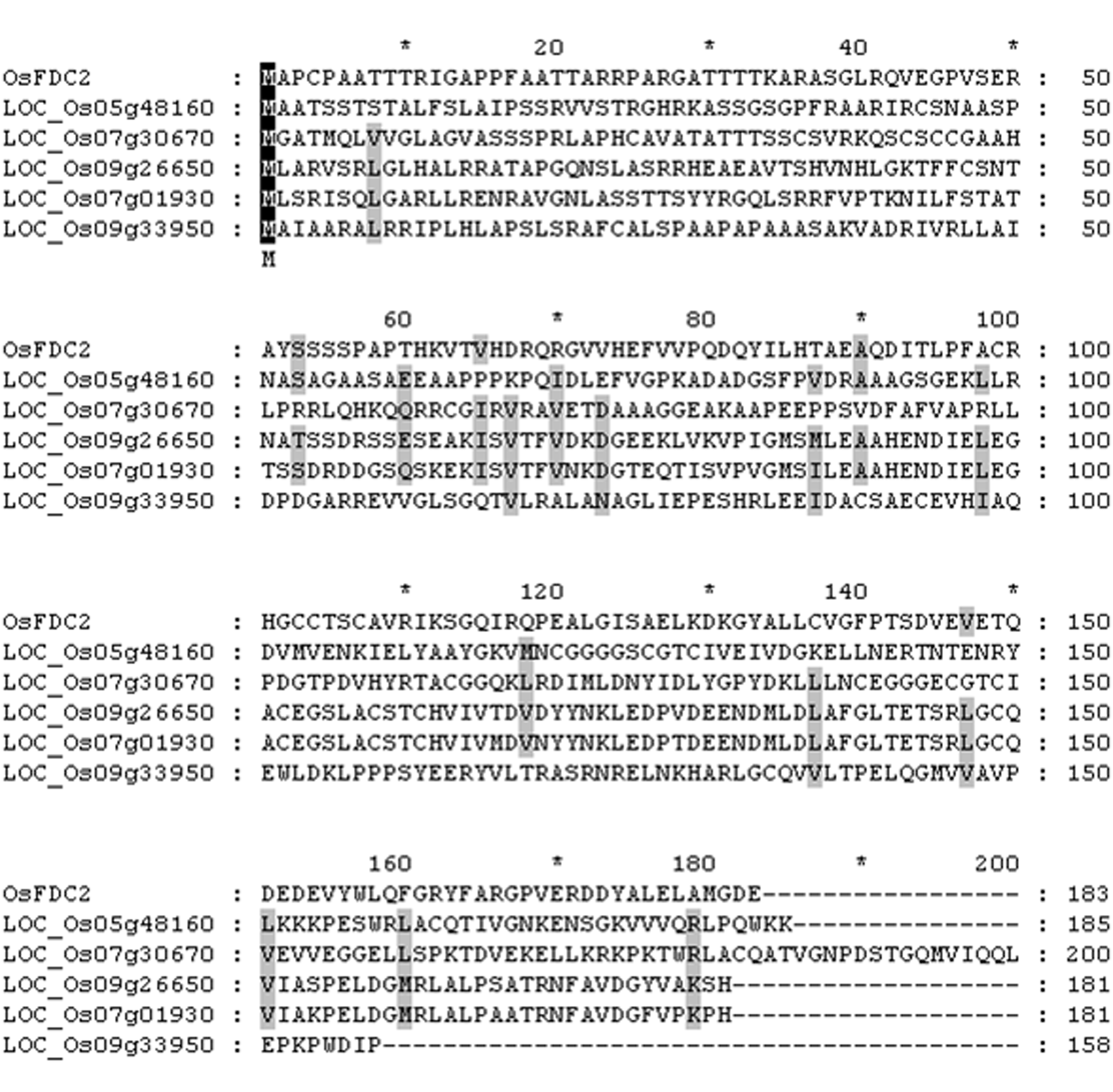

Supplement: S1 Fig — (TIF) [file pone.0143361.s001.tif]
