# Supplementary material for: Functional Inactivation of Putative Photosynthetic Electron Acceptor Ferredoxin C2 (FdC2) Induces Delayed Heading Date and Decreased Photosynthetic Rate in Rice
Source: PLoS One. 2015 Nov 24;10(11):e0143361. doi: 10.1371/journal.pone.0143361 (PMC4657970; doi:10.1371/journal.pone.0143361)
Supplement: S1 Table — (DOCX) [file pone.0143361.s002.docx]

| **Maker** | **Primers (5’ to 3’)** | |
| --- | --- | --- |
|  | **Sense** | **Anti-sense** |
| **ZJ8-2** | TGAAGACAAGTATGAGGCATG | ATTGAAAACGAAGGGATTATT |
| **ZJ8-4** | GAGTTACCTCCATCCTGTTGC | AGAGTCTTGATCTCGTGCTTC |
| **ZJ8-6** | TTAAAGCTAGTGCAAAAGGCA | CCATCGAAACTCTATTGAAAG |
| **ZJ8-35** | GGGTTTAGGTGAAGATGGTT | GTAAGCGCCCAGATTACATA |
| **ZJ8-39** | TCTATTGCAGTAGCACAATCT | CATGCCTCTATCCCAATAGC |
| **ZJ8-40** | GGTTAGATTTAGGCATTGGC | GCCCAACAACAGAACTTAG |
| **ZJ8-43** | ATCTGCTCCTTCCATCTTAAA | AGCAAGCTATTCGACAAGAG |
| **ZJ8-24** | TGGCACCAGCTCTTGATGAT | GCATGCCAATACAGGCAAGTT |
| **ZJ8-25** | CGCACCGATTTGATTTTCCCA | AAAAAGTTGGAAGTTCGTGTGT |
| **ZJ8-16** | GATATGGATTTTATCTCGTTT | ATGGAGGATGAAGATTAAGTA |
| **ZJ8-18** | ACACCATGTCGTTCGAGGTCC | AGGTTGCAATTTTGCATCAGG |
| **ZJ8-26** | TGGTCAAACGTTAAACACAG | AATTTACTCCTACATTCGGTG |
| **ZJ8-28** | TAGCCTACAATGCATGTGACCAA | AGGTGAAAGGAGAAGCGGCG |
| **ZJ8-17** | ACGAGTCGTTACGTTTTACGG | GCCTTGTTCGGAAGACCTACT |
| **ZJ8-8** | CCTCGGTTCTTTCAAAACGCA | GTCGGCTTCGTCGTCGGCGAC |
| **ZJ8-7** | CCGCTTCGAGACGACGCTGAG | AGCCCGAATCCCCAAAACCCT |
| **ZJ8-9** | GTGAACCAGCCACATGAGGGG | TCTTGCGGCGTGGGCGTAAAA |
| **ZJ8-10** | AAGGGTACTACATGGCATATC | CATATTAGCTTTCGATCAAAAT |
| **RM1350** | ATCAGCAAGAAAGCTCTGCTCC | AGGAAATTCGCCCTAGTAGATAG |

**S1 Table. Makers for map-based cloning of *OsFDC2*.**
